# Genetic Biomarkers in Chronic Myeloid Leukemia: What Have We Learned So Far?

**DOI:** 10.3390/ijms222212516

**Published:** 2021-11-19

**Authors:** Bilal Abdulmawjood, Beatriz Costa, Catarina Roma-Rodrigues, Pedro V. Baptista, Alexandra R. Fernandes

**Affiliations:** 1 i4HB—Institute for Health and Bioeconomy, NOVA School of Science and Technology, NOVA University Lisbon, 2819-516 Caparica, Portugal; b.abdulmawjood@campus.fct.unl.pt (B.A.); bv.costa@campus.fct.unl.pt (B.C.); catromar@fct.unl.pt (C.R.-R.); 2 UCIBIO—Applied Molecular Biosciences Unit, Department of Life Sciences, NOVA School of Science and Technology, NOVA University Lisbon, 2819-516 Caparica, Portugal

**Keywords:** chronic myeloid leukemia, Philadelphia chromosome, genetic biomarkers, miRNAs, genomic instability

## Abstract

Chronic Myeloid Leukemia (CML) is a rare malignant proliferative disease of the hematopoietic system, whose molecular hallmark is the Philadelphia chromosome (Ph). The Ph chromosome originates an aberrant fusion gene with abnormal kinase activity, leading to the buildup of reactive oxygen species and genetic instability of relevance in disease progression. Several genetic abnormalities have been correlated with CML in the blast phase, including chromosomal aberrations and common altered genes. Some of these genes are involved in the regulation of cell apoptosis and proliferation, such as the epidermal growth factor receptor (*EGFR*)*,* tumor protein p53 (*TP53*), or Schmidt-Ruppin A-2 proto-oncogene (*SRC*); cell adhesion, e.g., catenin beta 1 (*CTNNB1*); or genes associated to TGF-β, such as SKI like proto-oncogene (*SKIL*), transforming growth factor beta 1 (*TGFB1*) or transforming growth factor beta 2 (*TGFB2*); and TNF-α pathways, such as Tumor necrosis factor (*TNFA*) or Nuclear factor kappa B subunit 1 (*NFKB1*). The involvement of miRNAs in CML is also gaining momentum, where dysregulation of some critical miRNAs, such as miRNA-451 and miRNA-21, which have been associated to the molecular modulation of pathogenesis, progression of disease states, and response to therapeutics. In this review, the most relevant genomic alterations found in CML will be addressed.

## 1. Introduction

Chronic Myeloid Leukemia (CML) is a rare malignant proliferative hematopoietic disease with a global incidence of 1–2 cases per 100,000 adults [1]. In the United States alone, it is responsible for about 15% of leukemia diagnostics in adults [2]. In Europe, the median age at diagnosis is 60–65 years, being significantly lower within geographies with a younger population [3], but rare in children (the biomolecular aspects and treatment strategies in pediatric CML are reviewed elsewhere [4].

**Figure 1:**
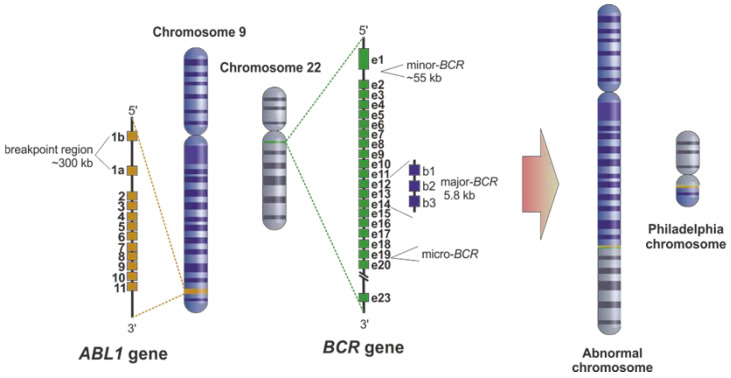
The origin of the Philadelphia chromosome. The Philadelphia chromosome results from the translocation between the Abelson murine leukemia (*ABL1*) gene on chromosome 9 with the breakpoint cluster region (*BCR*) gene on chromosome 22. Three break point regions might be involved in this translocation: (i) intron 13 or 14 of *BCR*, named major breakpoint (M-*BCR*), (ii) intron 1 of *BCR*, named minor breakpoint (m-*BCR*), and (iii) exon 19 of *BCR*, named μ breakpoint (μ-*BCR*). Concerning *ABL1*, the breakpoint usually involves the region between exons 1b and 2.

The Philadelphia (Ph) chromosome is the main molecular hallmark of CML, first discovered and described by Nowell and Hungerford (1960) [5]. The Ph chromosome arises from the fusion of the Abelson murine leukemia (*ABL1*) gene on chromosome 9 with the breakpoint cluster region (*BCR*) gene on chromosome 22 (Figure 1) [5]. This fusion leads to the expression of an oncoprotein called *BCR-ABL1*, characterized by a constitutively activated tyrosine kinase activity eliciting the cell inadequate differentiation, unrestricted replication, and resistance to apoptosis [6,7]. The enduring proliferation of these stem cells potentiates the occurrence of additional mutations, which often provide resistance to treatment, thus indicating a negative prognosis [8]. Most CML patients present a *BCR-ABL1* fusion downstream of the exons 13 and 14 of the *BCR* gene (Figure 1), originating mRNA transcripts with an e14 and/or an e13 junction, resulting in a 210 kDa chimeric protein-p210 *BCR-ABL1* (M-*BCR*) [9]. The second major fusion product is p190 *BCR–ABL1*, considered the smallest of the fusion proteins, which results from the minor break point region (m-*BCR*) (Figure 1) of the *BCR* gene producing the transcript e1a2. The p190 *BCR–ABL1* isoform is mainly present in Ph-positive acute lymphoblastic leukemia patients and rarely in CML. Still, when present, this isoform has been correlated with a more aggressive progression of the disease [9]. p230 *BCR-ABL1* is the third breakpoint cluster region (µ-*BCR*) occurring between *BCR* exons 19 and 20 [10,11]. This breakpoint has been commonly associated with neutrophilic-CML (CML-N) [12].

In terms of pathophysiology, CML is a myeloproliferative disorder that presents three phases, starting from a latent phase-chronic phase (CP), associated to leukemia stem cells (LSC), in which decontrolled clonal expansion of leukemia progenitor cells (LPCs) triggers the typical symptoms, such as splenomegaly, anemia, weight loss, fatigue, malaise, or pain in the upper left quadrant [13,14]. However, 50% of patients are asymptomatic and only diagnosed incidentally after routine laboratory tests [15]. Moreover, if the patient’s immune system is still competent, an asymptomatic status may be observable for over a long period [9]. If not tackled and treated, CML-CP spontaneously progresses to a more advanced and accelerated phase (CML-AP), when patients present more severe symptoms, including bone pain, skin infiltrate, lymphadenopathy, and intensification of anemia [6,9,16,17]. The final phase, called blast crisis phase (CML-BP), presents as an acute leukemia with aggravation of symptoms with fever, bleeding, and infections [6,18,19].

Tyrosine Kinase Inhibitors (TKIs) specifically designed to selectively hinder the kinase activity of the fusion oncoprotein, constitute the gold standard in CML treatment [20,21]. Imatinib (IM), a competitive inhibitor capable to bind to the ATP pocket in the kinase domain of *BCR-ABL1* protein, was the first TKI used in CML therapy, with over 95% of response rates [20,21]. The effectiveness of IM observed in all phases of CML, and therapy of most patients treated in CML-CP in clinical trials has resulted in a normal life expectancy [22,23] and population-based registries [24,25,26]. No serious toxicity has been shown after more than 20 years of use [27,28,29]. Deep molecular response (DMR) is commonly known as *BCR-ABL1* values of ≤0.01% IS and is described as different *BCR-ABL1* cutoff values, where molecular response 4 (MR4) is ≤0.01% IS, MR4.5 ≤ 0.0032% IS, and MR5 < 0.001% [30]. DMR was observed in more than 80% of patients (>70% continued in remission after discontinuation of treatment), which has prompted the proposal of discontinuation of treatment towards treatment-free remissions (TFR), thus reducing low-grade secondary effects, like muscle cramps and fatigue [31,32,33]. Owing to the diminished efficacy of IM in patients accumulating mutations within the fusion transcript, the second and third generations of TKIs, such as dasatinib, nilotinib, bosutinib, and ponatinib, were promoted [34]. In fact, the second generation TKIs (dasatinib, nilotinib, bosutinib) were developed to tackle the surge of mutations within the kinase domain linked to resistance to IM [35], which occurs in 4.6% of 1551 CP-CML patients over 10 years [23]. These TKIs result in more rapid responses compared to IM when used as a second-line treatment [36,37]. Third generation TKIs, such as ponatinib, were designed specifically to target the *BCR-ABL1-T315I* and compound mutations [38]. The T315I mutant form of *BCR-ABL1* lacks a threonine—T315 residue is the gatekeeper, when it is mutated to with an isoleucine (T315I), the entrance of a TKI inside the hydrophobic pocket is blocked, still allowing access to ATP [39]. Another example of the third generation TKIs is asciminib (ABL001), an allosteric inhibitor of the ABL-kinase, which exerts its effect by binding to the myristoyl and not the catalytic pocket of *BCR-ABL1* [40].

Genetic instability, characterized by the presence of a greater prevalence of mutations in the myeloid cell lineage, plays an important role in the progression of CML to advanced clinical phases [19]. Genomic instability induces changes to the DNA structure, such as increased frequencies of nucleobase mutation, microsatellite instability (MSI), and chromosome instability (CIN) [17]. The presences of chromosomal aberrations and small DNA mutations are thought to be associated with the progression of a relatively benign CML-CP to the aggressive CML-BP [41].

Looking at gene expression levels through CML development, the progression of chronic phase CML to advanced phase CML usually occurs in a two-step process, the first consisting of the evasion of cell differentiation and apoptosis, leading to enhanced expression of some genes implicated in the nucleosome, with activation of alternative signaling pathways and changes in cell adhesion [42]. In the second step, a relapse after initial successful treatment with imatinib may be associated with gene expression changes correlated to CML-AP, proposing that the process of progression persists in a subpopulation of CML cells during therapy [18,42]. *BCR-ABL1* fusion is the major therapeutic target for patients with CML and detection of changes in gene expression levels might help in the prognosis and diagnosis of CML [43]. While other genes have been reported as potential markers for prognosis and indicators of sensitivity/resistance to drugs in CML, including *TGF-β, TNF-α* [44], vascular endothelial growth factor A (*VEGFA*) [45], and 53 (*TP53*) [46,47,48,49,50] pathways. On the other hand, identification of genes whose expression is related with an increased probability of failure could be helpful in selecting more proper imatinib doses or drug combinations.

Epigenetic alterations (DNA methylation and/or histone modification) together with alterations of gene expression, namely by the expression of long noncoding RNAs (lncRNA) and microRNAs (miRNAs), have also been observed to be altered in CML [49,51,52]. Moreover, epigenetic dysregulation works in congruence with genetic abnormalities to provide an environment to enhance tumor formation, maintenance, and progression [49]. In stem cell disorders, such as CML, abnormal epigenetic regulation of some associated genes may have an essential part in the pathogenesis of the disease, and in the mechanisms of therapeutic responsiveness [50].

The following chapters will review the genetic characteristics of CML, starting by a chapter describing the variations found in the Philadelphia chromosome, followed by a description of the events occurring at the CML cell that result in genomic instability, with a resume of the studies that found common altered genes among CML patients, and molecular events associated with epigenetic regulation in CML. Finally, due to the relevance for CML progression and prognosis, the miRNAs mostly altered in CML will be discussed.

## 2. Variations of the Philadelphia Chromosome

Whereas 90% of CML patients exhibit the hallmark Ph chromosome, around 5–10% are Ph negative and might have variant abnormalities [53]. When detected, Ph chromosomes may involve 22q11 and one additional breakpoint (simple form) or in a complex form (involving 22q11, 9q34, and at least one additional breakpoint) [54]. Variations in Ph are due to breakpoints present in hotspots in the genome, generally in G-light bands, within the CG rich regions. The CG content has been shown to correlate with chromatin condensation and transcription activity, where open chromatin is transcriptionally active and, thus, more prone breakage and repair, and a concomitant tendency to recombination and translocation [55].

Cytogenetics is the gold standard for monitoring cytogenetic response and detecting the Ph chromosome. Karyotyping reports the number of Ph-positive metaphases out of at least 20 metaphases [56]. Furthermore, detection of additional chromosomal abnormalities is very important in the Ph-negative/positive clones (during therapy) especially in the case of clonal evolution, which may present in rare cases of development to myelodysplastic syndrome [57] or even to acute myeloid leukemia [58]. The prognostic impact of variant translocations has been reported [53,59,60,61,62,63,64,65,66]. Most studies indicate no difference to prognosis between standard and variant translocations [53,59,60,61,62,63], but there have been demonstrations of the association of Ph variations with poorer prognosis [64,65,66,67].

### 2.1. Ph-Positive Karyotype in CML

More than 90% of patients diagnosed with CML by cytogenetics presented the characteristic Ph chromosome, t(9;22) [68,69]. A recent study involving 250 Mexican Ph-positive CML patients [70] reported that 90.4% of patients expressed p210 *BCR-ABL1*, and just about 7% of patients with p210 *BCR-ABL1* expressed both isoform (b3a2/b2a2) [70]. However, only 5% of patients co-express p190/p210 *BCR-ABL1* fusions and those who co-express at least two or all p190/210/230 *BCR-ABL1* fusions had a poor prognosis [70].

### 2.2. Double Ph-Positive Karyotype in CML

Ph duplication is generally linked to over expression of *BCR-ABL1*, consequently linked to aggressive disease and deemed as an indicator of imatinib resistance [71,72]. This chromosome abnormality, coupled to the short isoform of *BCR-ABL1*, results in progression to CML-AP, deficiency of response to imatinib, and the necessity for a third generation TKI [73].

Vinhas and collaborators presented a patient with CML that initiated Imatinib with different dosages for several months and failed to induce a cytogenetic response [67]. After deeper analysis, the patient presented with a double Ph-positive cells and two isoforms of the gene: P210 (e14a2) and a rare P195 (e6a2) with no point mutations in the *ABL1* domain; while at the protein level, it showed *BCR-ABL1* expression and phosphorylation of ABL1 kinase domain, linked to the pathogenesis of CML [67]. The patient initiated bosutinib, and after monitoring attained a DMR to P210 and stable to P195 [67]. This study highlighted the importance of identification of more than one transcript to promote a more appropriate TKI regimen for improved response in CML patients [67].

## 3. Genomic Instability in CML

**Figure 2:**
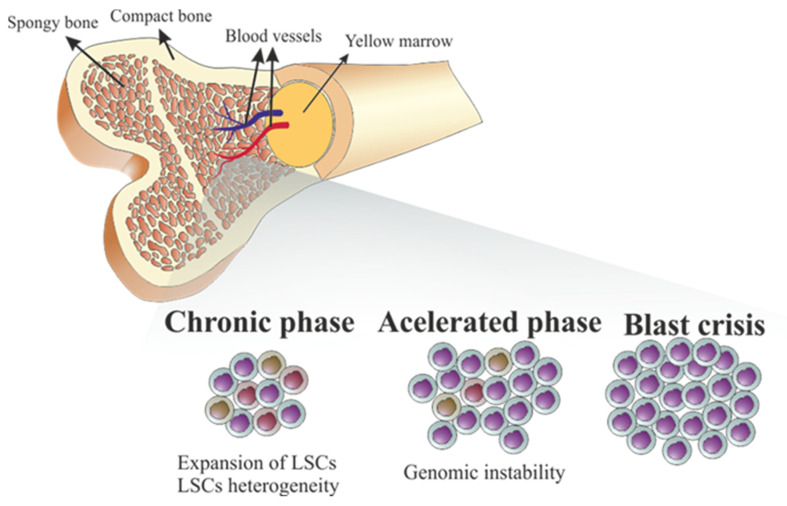
Stages of chronic myeloid leukemia (CML). The continuous activity of *BCR-ABL1* protein kinase induces a high proliferation of leukemia stem cells (LSCs) in bone marrow and blood vessels, resulting in a chronic phase. The appearance of resistance to tyrosine kinase inhibitors (TKI), together with a particular bone marrow microenvironment and accumulation of reactive oxygen species (ROS), induce bone marrow genomic instability in quiescent LSCs, that result in an accelerated phase that culminate in a blast crisis with worsening of symptoms.

The progression of CML from the CP into the BP is frequently accompanied by an increased genomic instability of the LSCs, resulting in microsatellite alterations, elevated telomerase activity, and cytogenetic instability [19,74,75,76]. It is now generally accepted that LSCs are a heterogeneous group of cells, with TKI-resistant quiescent LSCs found at the bone marrow microenvironment [76,77]. It is likely that interaction of these quiescent LSCs with surrounding stromal cells located in the bone marrow niche, favors genomic instability, and further progression into the BP (Figure 2) [76,78].

This genomic instability has been associated to an aberrant response to DNA damage, which, in the case of CML, seems to result from the increased concentration of reactive oxygen species (ROS) induced by the activity of *BCR-ABL1* kinase in the bone marrow microenvironment (Figure 2) [78,79,80]. Although the mechanism of ROS formation in CML cells is not entirely understood, it is postulated that it might result from modifications in the mitochondrial membrane potential induced by the Rac2-MRC-cIII GTPase activity in *BCR-ABL1* positive cells [79]. The increased production of the superoxide anion (**·** $O_{2}^{-}$) in CML cells potentiates single-strand DNA damage (e.g., via the formation of 8-oxoguanine) or double-strand DNA breaks [81,82]. While oxidative DNA damage is, in a normal cell, corrected by DNA repair mechanisms (e.g., homologous recombinant repair, non-homologous end-joining, single strand annealing, etc.), in CML cells, the accuracy and fidelity of these repair mechanisms are compromised due to ROS accumulation [19,81]. Furthermore, the higher telomerase activity that leads to telomere shortening and the higher proliferation rate of CML cells due to the loss of function in proteins associated to checkpoint control further contribute to this genomic instability [83,84].

Although the genomic instability due to ROS-induced DNA lesions might occur randomly, there are common genetic abnormalities found in CML cells at the chromosomal level (e.g., trisomy 8, isochromosome 17, loss of chromosome Y, monosomy 7, duplication of Ph, duplication of chromosomes 19, 21 or 17) [85,86] or at the gene level (Table 1). Recently, an integrated computational biology analysis identified the most common genes altered in CML patients by using a scoring methodology that included if there were multiple studies supporting the correlation of that gene with CML; if that gene is involved in high number of CML-related molecular pathways, if that gene presented any type of biological connection with other CML candidate genes [47]. This scoring allowed the identification of 9 genes relevant to CML: *EGFR*, *TP53*, catenin beta 1 encoding gene (*CTNNB1*), janus kinase 2 encoding gene (*JAK2*), *TNF*, *ABL1*, *VEGFA*, B-cell lymphoma 2apoptosis regulator encoding gene (*BCL2*), and Schmidt-Ruppin A-2 proto-oncogene (*SRC*) [47]. Moreover, they also found nine additional genes that were present in multiple CML-pathways with strong connections with other CML-related genes but very few descriptions in the literature, including: transforming growth factor beta 1 (*TGFB1*), transforming growth factor beta 2 encoding gene (*TGFB2*), protein kinase 2 beta encoding gene (*PTK2B*), protein kinase 2 encoding gene (*PTK2*), AKT serine/threonine kinase 1encoding gene (*AKT1*), interleukin 1 beta encoding gene (*IL1B*), mitogen-activated protein kinase 1 encoding gene (*MAPK1*), Fyn proto-oncogene, Src family tyrosine kinase encoding gene (*FYN*), platelet-derived growth factor receptor encoding gene, beta polypeptide encoding gene (*PDGFR*-β), and mitogen-activated protein kinase 3 encoding gene (*MAPK3*) [47].

Recently, FISH and Next-Generation Sequencing (NGS) allowed studying of the correlation between expression, copy number, and activity of *BCR-ABL1* with the mutational status, copy number, and expression of telomere maintenance genes in five *BCR-ABL1*-positive cell lines (K562, KU-812, LAMA-84, MEG-A2 and MOLM-1) [46]. Although no pathogenic variations were found in telomere-associated genes, they identified mutations and/or copy number variations in tumor suppressor genes, namely in *TP53* (loss and mutations), *CDKN2A* (deletion), *ATM* (mutations) [46]. In line with previous studies [83], a correlation between expression of *BCR-ABL1* and telomere length was found [46]. The importance of *TP53* mutation or loss of function, including codon 72 polymorphism, was previously described as being preponderant for CML progression and therapy resistance [48,49,50,89,90,91,92]. Supporting NGS data analysis showed that deletion of *CDKN2* and mutations in ATM and homologue gene ATR results in CML progression and increased genomic instability [93,94,95].

The protection from the bone marrow microenvironment together with intrinsic characteristics make some LSCs resistant to chemotherapeutics [96]. These resistant LSCs can propagate after therapeutic discontinuation, prompting a blast crisis [96]. A single cell transcriptomic analysis allowed to identify genes associated with proliferation in *BCR-ABL1* positive LSCs at diagnosis (e.g., Mechanistic Target Of Rapamycin Kinase (*MTOR*) encoding gene, E2F targets genes, G2M checkpoint genes, and genes involved in oxidative phosphorylation and glycolysis). Moreover, an altered expression of *CLU*, *FCER1A*, *GAS2*, *MZB1*, *RGS2*, and *CXCR4* in LSCs relative to normal hematopoietic stem cells (HSCs) and *BCR-ABL1* negative cells was found [44]. This transcriptomic analysis also allowed the identification of genes expressed in LSCs from good TKI responders compared to poor TKI responders [44]. They found that good TKI responders had an enrichment of genes and pathways involved in increased proliferation, such as c-*MYC*, *E2F* and G2M-checkpoint genes, while poor TKI responders presented an upregulation of genes associated with the TGF-β and TNF-α pathways, along with a quiescence signature [44]. This single cell transcriptomic analysis also allowed to get insights into the genomic traits of resistant quiescent LSCs relative to normal HSCs, which included the overexpression of genes involved in TGF-β signaling (e.g., *SKIL*), regulators of NF-kB (*NFKB1A* and *SQSTM*), *HIF1A*, *WT1*, *WTAP*, in Wnt/β-catenin pathway (e.g., *GAS2* and *CTNNB1*), and downregulation of chemokine receptor *CXCR4* and *FOS* [44,96]. Overall, three specific pathways (*TGF-β, TNF-α* and *JAK-STAT*) and two genes (*CTNNB1* and *NFKB1A*) were shown to be involved in TKI resistance of quiescent LSCs relative to quiescent normal HSCs [44].

Previously, Villuendas and collaborators identified a set of 46 genes with altered expression in patients that respond to imatinib, compared to patients that did not respond; it included genes involved in drug metabolism (prostaglandin-endoperoxide synthase 1, *PTSG1*), genes involved in cell adhesion (tenascin C, *TNC* and sorbin and SH3 domain containing 3, *SORBS3*) or genes encoding protein kinases and phosphatases (Bruton tyrosine kinase, *BTK* and protein tyrosine phosphatase non-receptor type 22, *PTPN22*) [88].

The knowledge of alterations in the expression of genes in CML has inspired several studies to improve the CML diagnosis and therapeutics. The potentiality in the application of other sets of genes for CML molecular screening might allow a more precise management towards a personalized medicine and a proper treatment of patients with diagnostically difficult cases of CML [97]. For example, the presence of VEGFR21416A>T and VEGFA 936C>T polymorphisms on vascular endothelial growth factor receptor encoding gene (*VEGFR2*) might be correlated with poorer prognosis and TKI resistance [45]. Several other studies analyzed the potential of using *TP53* as a biomarker for CML susceptibility, therapeutical response, and clinical outcome [48,49,50]. The knowledge of the importance of this gene in CML progression goes back to 1998 when two independent studies reported a common deletion at 17p13.3 in leukemia [89] and molecular alterations in the *TP53* gene in cells from CML patients [90]. The central role of P53 in tumor suppressor responses, such as senescence, apoptosis, cell-cycle arrest, or modulation of autophagy gives this protein the title of “guardian of the genome” [98,99]. Mutations in *TP53* are frequently found in tumors, rendering cancer cells advantages to proliferate and thrive, even in stressful conditions found in a tumor microenvironment [100,101]. The relevance of *TP53* mutations in CML was emphasized via the description of the presence of intronic SNPs correlating to CML progression and TKI response [102]. In another study, Weich and collaborators identified the c.213 G>C polymorphism in *TP53*, which correlated with a worse clinical outcome [50].

Keeping in mind targeted gene therapy, RITA (also known as NSC652287) was used to bind to p53 and block its degradation, together with CPI-203, a bromodomain and extra terminal protein (BET) inhibitor that disrupts chromatin-dependent signal transduction to target *c-MYC* [103]. The application of this dual targeting resulted in the near elimination of transplantable human LSCs in mice, suggesting a new strategy to eradicate LSCs [103].

Neviani and collaborators showed that *BCR-ABL1* expression was able to trigger the downregulation of the tumor suppressor protein phosphatase 2A encoding gene (*PP2A*), together with the expression of genes involved in the *JAK2/β-catenin* pathway in quiescent LSCs relative to normal HSCs. The application of PP2A-activating drugs (PADs, such as FTY720) suppressed the *JAK2/β-catenin* pathway via *PP2A* with consequences to quiescent LSCs survival, suggesting the application of PADs to improve the prognosis in TKI-refractory CML [104].

## 4. Epigenetic Regulation in CML

**Figure 3:**
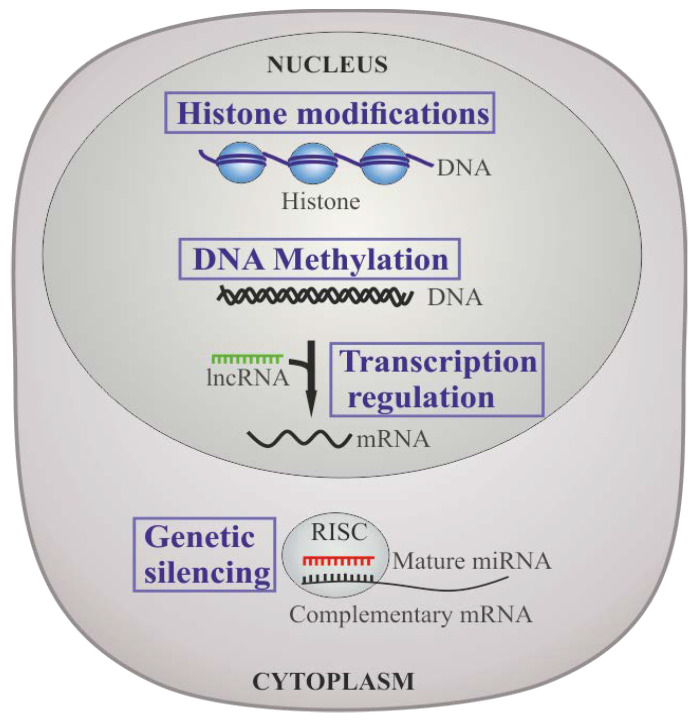
Epigenetic regulation in CML. Histone modification is pivotal in gene regulation. Generally, DNA methylation exerts a negative modulation of gene transcription through DNA methyltransferases (DNMTs) and methyl-cytosine dioxygenases at cytosine residues in CpG dinucleotides. Non-coding RNAs play crucial roles in regulating gene expression, either via miRNA-induced silencing complex (miRISCs), targeting mRNA for degradation; or lncRNAs which are involved in regulation of gene transcription, epigenetic modifications, and posttranscriptional processing.

The epigenetic status of a cell might be controlled by different mechanisms, namely DNA methylation, histone modification, and post-transcriptionally via non-coding RNAs [51]. These mechanisms may exhibit effects at the DNA level by altering DNA conformation and availability for transcription, damage, and/or repair; at the RNA level by altering RNA expression levels and stability, and at the protein level by regulating protein translation or post-translational protein modifications (Figure 3) [51].

DNA methylation plays an essential role in chromatin organization and gene expression regulation, which is the most described epigenetic mechanism [105]. DNA methylation consists of the suppression of gene transcription mediated by DNA methyltransferases (DNMTs), that can be passively removed throughout the cell division process or actively removed by methyl-cytosine dioxygenases [106]. This action takes place mostly at cytosine residues in CpG dinucleotides [106]. Hypermethylation of CpG islands may lead to inactivation of tumor suppressors and other genes, especially in promoter regions; other genes, such as oncogenes, are usually hypomethylated [52]. This phenomenon plays a central role in solid tumors and leukemias [107]. The effects of dysregulation of DNA methylation in the survival pathways of CML by epigenetically dysregulated genes have been highlighted in several studies. Firstly, *MTSS1* is a tumor suppressor that is mainly downregulated in both LSC and progenitor cells of CP patients and in CML mouse models [108]. However, during TKI therapy, it is retained to normal levels [108]. It is likely that inhibition of *MTSS1* results in promotion of CML and progenitor cells proliferation and motility, which are decreased upon MTSS1 increased expression [108]. Secondly, *SIRT1* upregulation in CML occurs by hypermethylation of the *HIC1* gene—a repressor that acts in congruence with *SIRT1* to auto-regulate *SIRT1* expression [107]. The *HIC1* promoter is hypermethylated in CP, which increases during the progression of the disease [109,110]. Finally, the CD70/CD27 axis has been demonstrated to activate non-canonical Wnt signaling in the presence of TKI, taking part in LSC survival [111].

Histone modifications are essential in gene regulation involving transcription [112]. Histone modifications mainly comprise of acetylation, methylation, phosphorylation [112], and different posttranslational modifications [113]. Several studies have established that the activation of oncogenes and inactivation of tumor suppressor genes is associated in the pathogenesis of CML [114]. The loss-of-function mutations of the CML-related genes are correlated to the dynamic changes of histone modifications [115,116,117]. For instance, promoter histone hypoacetylation results in *PDH1* silencing [118] and consequent decrease of mRNA and protein levels of *BCR-ABL1* in CML and LAMA-84 cells [119]. However, hyperacetylation stimulates the expression of p21 and/or p27 [52].

Non-coding RNAs are important players in gene expression regulation at the level of transcription, RNA processing, and translation [120,121,122,123,124]. They act in several gene regulation processes that may end up in complex diseases, such as cancer [125]. The first suggestion that miRNAs are correlated to cancer was in 2002 [126,127], and since then miRNAs have been identified as essential switches in cancer pathology [128]. Moreover, deregulated miRNAs have oncogenic and/or tumor suppressive functional roles [129]. In CML, miRNAs have been shown to act both as oncogenes and tumor suppressor genes [129,130,131,132,133]. Since circulating miRNAs can be detected in body fluids, this makes them ideal biomarkers for tumor diagnostics and progress monitoring [134]. Numerous long non-coding RNAs (lncRNAs) have been discovered recently and recognized as essential in gene regulation [133]. lncRNAs are >200 nt in length, transcribed via RNA polymerase II/III, and weakly conserved [134]. It has been identified that lncRNAs may act as signals, guides, decoys, and scaffolds in the gene expression regulation of every step of the gene regulatory network [135,136,137,138,139], including in gene transcription, epigenetic modifications (such as chromatin and protein modification), and posttranscriptional processing (splicing, stability or translation of mRNAs) [134]. Different studies demonstrated that some lncRNAs can be the hallmark of diverse human cancers [140,141,142,143]. In CML cells, a comprehensive analysis of lncRNAs was applied and a novel lncRNA lncRNA-BGL3 was discovered to act as a key regulator of *BCR-ABL1*-mediated cellular transformation [144,145].

## 5. miRNAs Dysregulation in CML

**Figure 4:**
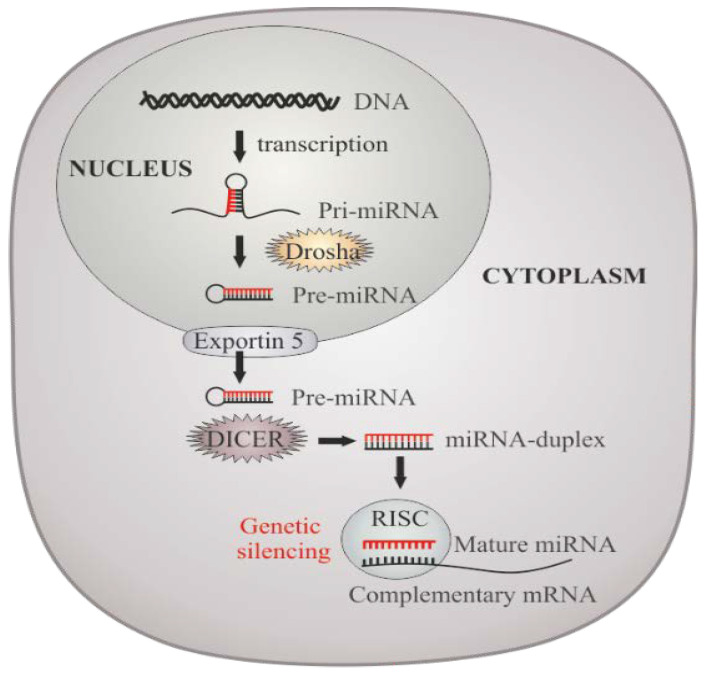
miRNAs biogenesis and molecular processing/action. Pri-miRNAs are synthesized in the nucleus, recognized and cleavage by Drosha into a smaller stem-looped structure–the pre-miRNA. Pre-miRNAs are then transported into the cytoplasm for further processing by Dicer, originating the mature miRNAs, which will integrate the miRISC. Genetic silencing or block of translation will depend on the level of homology between miRNAs and their complementary target sequences primarily located within 3′-UTRs of mRNAs.

miRNAs are short sequences (~20–23 nucleotides) of conserved non-coding RNAs that take part in the translational regulation of several genes and are involved in the regulation of important biological activities by selectively binding to their mRNA transcripts, blocking translation, and protein expression (Figure 4) [47,146,147]. It has been estimated that miRNAs control approximately 60% of the transcriptome and play important roles in most of cellular processes like proliferation, differentiation, development, cell fate determination, and apoptosis [148].

Regulation of tumorigenesis in CML is a highly complex process, where different players take part as described above, and among those crucial players, several miRNAs have been identified as dysregulated (Table 2) [149].

Among the miRNAs deregulated in CML, the expression profile of some of them have been associated to patients’ response to TKIs (e.g., imatinib) (Table 2) [128]. For instance, there is an increased expression of miRNA-26a, miRNA-29c, miRNA-130b, and miRNA-146a in patients that respond to TKI treatment compared to non-responsive patients [128]. This difference could be attributed to the downregulation of their potential targets (e.g., BIRC2 baculoviral IAP repeat containing 2 (cIAP1) and MCL1 apoptosis regulator (MCL1)), important in tumor cell survival following TKI treatment. Interestingly, the up-regulation of miRNA-23a, miRNA-30a, miRNA-30e, miRNA-203, miRNA-320, and miRNA424, known to target *BCR-ABL1* is also adding benefits to TKI responsive patients [128]. Furthermore, the levels of miRNA-451, could constitute an excellent biomarker to predict a response to TKI [106] and its expression was associated with a good prognosis as it also targets *ABL* and *BCR-ABL1* directly [128].

In the following two sections, we will highlight the most relevant miRNAs, beginning with miRNA-451, as a representative of tumor suppressor miRNAs, by targeting the fusion mRNA *BCR-ABL1*, and with miRNA-21, a relevant onco-miRNAs in solid and hematological tumors [149].

### 5.1. miRNAs That Target BCR-ABL1

As *BCR-ABL1* is the hallmark of CML, miRNAs that target this mRNA might constitute vital biomarkers for CML treatment and/or diagnostics [149]. For example, several studies demonstrated that miRNA-451 behaves as a tumor suppressor in many malignancies, such as hepatocellular carcinoma (HCC), glioblastoma, gastric cancer (GC), and non-small-cell lung carcinoma (NSCLC), as it is often downregulated [165,166]. Additionally, publish data correlated its expression levels with disease stage (e.g., metastasis) and shorter overall survival [165,167].

In 2012, Scholl, Hassan, and Zalcberg reported an interesting case where patient harboring IM-resistant G250E mutation achieved a complete cytogenetic response opposed to patients with the IM-sensitive M351T mutation, who progressed to CML-AP and died. This unexpected outcome was explained by the very low miRNA-451 expression level of patients with IM-sensitive M351T mutation in comparison with patients with G250E mutation [162]. The potential use of miRNA-451 to predict TKI response with good sensitivity/specificity was further supported by Alves and co-workers (2019), who developed a predictive model for an optimal response after one year of TKI treatment based on the expression levels of miRNA-21 and miRNA-451 at diagnosis [149].

More recently, a microarray analysis using plasma of CML patients versus healthy individuals clearly indicated a lower expression level of miRNA-451 in the former. Additionally, it was shown that expression levels decreased from CML-CP to advanced phase CML-AP and ultimately to CML-BP [159]. This was later corroborated by the analysis of miRNA-451 expression in the leukocytes of CML patients, where it was shown that miRNA-451 is downregulated at the time of diagnosis, compared with healthy controls and in patients in hematological relapse, in divergence to normal or rather increased levels in patients with a major molecular response (MMR) and suboptimal response after IM treatment [168]. As its potential target is *BCR-ABL1*, the authors proposed the existence of a reciprocal regulatory loop between them, and that *BCR-ABL1* inhibits miRNA-451, its negative regulator, to increase its expression and allow CML progression [168]. This regulatory loop might be the basis of the up-regulation of miRNA-451 in CP CML patients who achieved MMR after IM treatment, compared to recently diagnosed CML-CP patients [161]. Moreover, miRNA-451 expression levels were also correlated with IM response, as its levels were downregulated in IM-resistant patients compared to IM-responsive or healthy controls [161]. Nevertheless, no significant variations were observed between IM-responsive and healthy control groups [161].

Also, two recent studies showed that *c-MYC* oncogene is also a target of miRNA-451, which may also explain its role in CML tumorigenesis and IM resistance. Indeed, Liu and collaborators showed c-*MYC* was up-regulated in IM-resistant cells and that miRNAs-144 and miRNAs-451 were simultaneously downregulated and that restoring miRNA-144/451 expression could induce IM-resistant cells apoptosis [163]. These results agree with the fact that overexpression of miRNA-451 increased cisplatin sensitivity in lung cancer and sensitized colorectal and gastric cancer cells to radiotherapy [165].

Besides miRNA-451 highlighted above, other miRNAs, such as miRNA-29b, miRNA-138, and miRNA-203, fall into this category and will be addressed together with another tumor suppressor miRNA relevant for CML phenotype, the miRNA-486-5p. The expression of miRNA-29b is downregulated in CML patients, which is explained by its ability to silence several oncogenes, such as antiapoptotic myeloid cell leukemia sequence 1 (*MCL*-1) and p53 inhibitors [153]. Imposed expression of miRNA-29b decreases *BCR-ABL1* mRNA as well as *BCR-ABL1* protein expression and inhibited K562 cell proliferation, leading to an increased apoptosis [153]. Additionally, *BCR-ABL1* expression is also regulated by miRNA-138, a tumor suppressor miRNA downregulated in CML patients [156]. Overexpression of miRNA-138 results in the downregulation of the fusion protein, which explains why miRNA-138 expression levels are restored after IM treatment [156]. Furthermore, overexpression of this miRNA induces inhibition of proliferation, cell cycle arrest, and increases IM-induced apoptosis [156].

miRNA-203 is silenced in CML due to genetic and epigenetic (promoter CpG hypermethylation) mechanisms [157], where a correlation between hypermethylation and decrease expression of miRNA-203 in *BCR-ABL1* positive CML cell lines but not in hematopoietic neoplasms without *ABL1* alterations [157]. Microarray analysis showed that IM up-regulates the epigenetically silenced tumor suppressor miRNA-203 by inducing the demethylation of its promoter region in CML cells [158]. The increased expression of miRNA-203 was concomitant with the lower expression levels of *BCR-ABL1* and reduced proliferation of CML cells [158]. Reintroduction of miRNA-203 in cells containing the *BCR-ABL1* T315I mutation, which is refractory to imatinib, not only significantly inhibited cell growth, but also increased sensitivity to the TKI [169].

Early this year, the potential role of miRNA-486-5p as a prognostic biomarker for CML was put forward by assessing miRNA-486-5p expression levels in K562 cell line and in peripheral blood leukocytes of CML patients before and after imatinib treatment [164]. Data showed the onco-suppressor role of miRNA-486-5p, which is downregulated in both CML-CP patients and the CML cell line [164]. The same study correlated higher miRNA-486-5p expression levels at diagnosis with better prognosis and faster achievement of complete hematologic response to imatinib treatment.

### 5.2. miRNA-21

As mentioned above, miRNA-21 expression levels at diagnosis assisted the establishment of a model to predict CML patient’s response after one year of TKI treatment [149]. A correlation between low levels of this miRNA at the time of CML diagnosis and an optimal response to TKI therapy was observed, which constitutes an opposite result to the one obtained for miRNA-451 [149]. The inverse correlation between these two miRNAs expression levels is also observed in TKI resistant CML cell lines, where miRNA-21 is upregulated and, by opposition, miRNA-451 is downregulated [149]. These results, which reveal the potential of miRNA-21 as a novel CML biomarker, agree with the observation that miRNA-21 is one of the most upregulated miRNAs in cancer, targeting several tumor suppressor genes implicated in apoptosis, proliferation, and invasion, and thus contributing to tumorigenesis [150]. Also, some reports state that this miRNA-21 acts as an anti-apoptotic and pro-survival factor and is implicated in chemotherapy resistance [150,151]. In addition, the expression level of miRNA-21 is increased in CML patients at the time of diagnosis in comparison with the healthy control group [151]. As noted above for miRNA-451, there seems to exist a correlation between miRNA-21 expression levels at diagnosis and disease stage, indicating that they are higher at CML-BP, and decrease from CML-AP to CML-CP [151]. These results suggest that miRNA-21 is involved in CML progression and can be used as a biomarker to complement diagnosis and to assess disease progression [151]. Authors also concluded that imatinib decreases miRNA-21 expression, thus being able to be a useful biomarker to monitor treatment response [151]. Similar results were reported in the context of glioblastoma, in which miRNA-21 inhibition leads to caspase activation and apoptosis [170]. The involvement of miRNA-21 overexpression in drug resistance is associated with a reduction in chemotherapy-induced apoptosis because of miRNA-21 ability to decrease the expression of many genes implicated in this programmed cell death, such as *PDCD4* and *PTE* [152]. Li and collaborators (2010) studied themiRNA-21 inhibition through specific anti-miRNA-21 oligonucleotide (AMO-miRNA-21) [171]. Silencing this miRNA resulted in G1-phase arrest, growth inhibition, and apoptosis, and authors concluded thatAMO-miRNA-21 sensitized a CML cell line to arsenic trioxide (ATO), possibly by apoptosis promotion due to upregulation of *PDCD4* [171]. According to Bai and co-workers, downregulation of *PTEN* and consequent increase in AKT activity by miRNA-21 overexpression contributes to CML cells resistance to daunorubicin [172]. The author’s results reveal that one of the mechanisms by which miRNA-21 acts in drug resistance comprises the PI3K/AKT pathway mediated through downregulation of *PTEN* expression [172]. Furthermore, miRNA-21 expression is also implicated in resistance to several drugs in different types of cancers, such as gemcitabine in pancreatic cancer, cisplatin in stomach cancer, doxorubicin in bladder malignancy, and EGFR-TKI in NSCLC [150].

Disruption of apoptosis does not seem to be the only mechanism by which miRNA-21 is involved in drug resistance. Seca and collaborators reported that downregulation of this miRNA is accompanied by the decreased expression of Bcl-2 protein, and that inhibition of miRNA-21 with anti-miRNAs increased autophagy-related proteins, such as Beclin-1, Vps34, and LC3-II, thus increasing sensitivity to doxorubicin and etoposide [152]. This observation indicated that miRNA-21 might modulate CML sensitivity to chemotherapy-induced autophagy [152].

Other studies have addressed the autophagy role in CML and demonstrated that this catabolic pathway allows imatinib-treated CML cells to evade cell death [154,155]. Yu and collaborators demonstrated that upregulation of miRNA-30a leads to downregulation of the pro-autophagic proteins Beclin 1 and autophagy protein 5 (ATG5) and, consequently, autophagy downregulation, thus increasing imatinib-induced cytotoxicity and promoting mitochondria-dependent intrinsic apoptosis [154]. Recently, Khalil and co-workers reported that miRNA-30a is upregulated in imatinib-responders compared to imatinib-resistant patients, which confirms that decreasing autophagy benefits imatinib-induced cytotoxicity [155]. Additionally, the authors were able to negatively correlate miRNA-30a level with Sokal score and *BCR-ABL1* following treatment [155].

### 5.3. miRNAs Role in Resistance to TKIs and Blast Crisis Progression

The role of mir-21 in TKIs resistance in CML is further supported by Wang and colleagues’ report that mir-21 inhibition by antagomiR-21 sensitized LSCs to IM treatment by increasing imatinib-induced apoptosis [173,174]. In addition to mir21, miR-30a is also reported to be associated with TKIs’ inability to eliminate quiescent LSCs [173]. Yu and collaborators established the role of autophagy in LSCs drug resistance through assessment of miR-30a expression levels and Beclin 1 mRNA levels. In comparison with CD34^−^ cells, and despite IM treatment, LSCs exhibited lower levels of miR-30a and increased mRNA levels of Beclin 1, which uncovers that targeting miR-30a-mediated Beclin 1 expression, may be crucial to eliminate CML LSCs and prevent disease relapse [154].

The interaction between LSCs and stromal cells present in the bone marrow niche where they reside is well established and seems to support LSCs maintenance in bone marrow despite TKI treatment [174]. Although miR-126 was described as a pro-leukemic miRNA in acute myeloid leukemia (AML), *BCR-ABL1* was demonstrated to downregulate miR-126 in CML. These conflicting observations are explained by Zhang and colleagues’ report that endothelial cells supply miR-126 to CML LSCs and contribute to LSCs quiescence and self-renewal. The TKIs inhibition of *BCR-ABL1* further increases miR-126 levels, which contribute to LSCs persistence [175].

The progression of CML from the CP into the BP is often associated with altered miRNA expression of the LSCs. According to Eiring and collaborators, the downregulation of miR-328 by *BCR-ABL1* is observed in CML-BP cells but not in CML-CP cells and supports CML progression to BP through differentiation arrest [176]. Reduced levels of miR-328 allow for the inhibitory interaction between the translational regulator heterogeneous ribonucleoprotein E2 (hnRNP E2) and CCAAT/Enhancer-binding Protein α (C/EBPα), which promotes blasts differentiation and compromises survival [128,176]. Furthermore, progression from CP to BP is accompanied by downregulation of 15a, miR-15b, and miR-16, and consequently increased levels of their target oncogenes B lymphoma Mo-MLV insertion region 1 homolog (Bmi-1) and Bcl-2, which promote survival and proliferation [177].

## 6. Conclusions

Recent reports in the literature have highlighted the need to detail the pathways causing genomic instability in CML, since these are likely to identify additional molecular players that may be considered candidates for targeted therapeutic intervention to overcome drug resistance. Moreover, identification of the main genes and pathways altered in CML opens new doors to elucidate the complexity of this liquid tumor and may identify new biomarkers to improve early detection/diagnosis, and personalized treatment, via the development of targeted therapies. miRNAs involvement in CML is a promising research area not only to find new biomarkers to incorporate in diagnosis and to assess disease progression and treatment response but also for the development of innovative therapy regimens.

## Figures and Tables

**Table 1:** Genes whose expression is altered in CML.

Gene (Protein)	Description	NCBI Gene ID	Protein Function	CML Analysis	References
***ABL1* (ABL1)**	ABL proto-oncogene 1, non-receptor tyrosine	25	Cell division, adhesion, differentiation, and response to stress	Chronic phase Blast crisis	[47]
***AKT1* (AKT1)**	AKT serine/threonine kinase 1	207	Regulation of cell proliferation, survival, metabolism, and angiogenesis	Chronic phase Blast crisis	[47]
***ATM* (ATM)**	Ataxia-telangiectasia mutated serine/threonine kinase	472	Cell cycle checkpoint	Blast crisis	[44]
***BAP1***	BRCA1 associated protein 1	8314	Regulation of cell cycle and growth	Chronic phase Blast crisis	[87]
***BCL2* (BCL2)**	BCL2 apoptosis regulator	596	Regulation of apoptosis	Chronic phase Blast crisis	[47]
***BTK* (BTK)**	Bruton tyrosine kinase	695	B-cell development	TKIs^1^ resistance	[88]
***CDKN2A***	Cyclin dependent kinase inhibitor 2A	1029	Regulation of cell cycle progression	Blast crisis	[44]
***CLU* (CLU)**	Clusterin	1191	Regulation of apoptosis and cell proliferation	Chronic phase Blast crisis	[44]
***C-MYC* (c-MYC)**	MYC proto-oncogene, bHLH transcription factor	4609	Regulation of cell cycle progression, apoptosis, and cellular transformation	Good response to TKIs ^1^	[44]
***CTNNB1* (CTNNB1)**	Catenin beta 1	1499	Cell adhesion	Chronic phase Blast crisis TKIs ^1^ resistance	[44,47]
***CXCR4* (CXCR4)**	C-X-C motif chemokine receptor 4	7852	Regulator of apoptosis, calcium-mediated signaling, response to cytokine stimulus	Chronic phase Blast crisis TKIs ^1^ resistance	[44]
***E2F* (E2F)**	E2F transcription factor 1	1869	Cell cycle regulation	Good response to TKIs ^1^	[44]
***EGFR* (EGFR)**	Epidermal growth factor receptor	1956	Cell proliferation	Chronic phase Blast crisis	[47]
***FCER1A* (FCER1A)**	Fc fragment of IgE receptor 1a	2205	Alpha subunit of immunoglobulin E involved in allergic response	Chronic phase Blast crisis	[44]
***FOS* (FOS)**	Fos proto-oncogene, AP-1 transcription factor	2353	Regulation of cell proliferation, differentiation, and transformation	TKIs ^1^ resistance	[44]
***FYN* (Fyn)**	FYN proto-oncogene, Src family tyrosine	2534	Regulation of cell growth	Chronic phase Blast crisis	[47]
***GAS-2* (GAS-2)**	Growth arrest specific 2	2620	Apoptosis	Chronic phase Blast crisis TKIs ^1^ resistance	[44]
***HIF1A* (HIF1A)**	Hypoxia inducible factor 1 subunit alpha	3091	Response to hypoxia	TKIs ^1^resistance	[44]
***IL1B* (ILB1)**	Interleukin 1 beta	3553	Cytokine involved in inflammatory response, cell proliferation, differentiation, and apoptosis	Chronic phase Blast crisis	[47]
***JAK2* (JAK2)**	Janus kinase 2	3717	Regulation of cell growth, development, and differentiation	Chronic phase Blast crisis	[47]
***KDR* (VEGFR)**	Vascular endothelial growth factor receptor	3791	Proliferation and migration of vascular endothelial cells	Mutations correlate to poor prognosis	[45]
***MAPK1* (MAPK1)**	Mitogen-activated protein kinase 1	5594	Regulation of proliferation, differentiation, transcription, and development	Chronic phase Blast crisis	[47]
***MAPK3* (MAPK3)**	Mitogen-activated protein kinase 3	5595	Regulation of proliferation, differentiation, and cell cycle progression	Chronic phase Blast crisis	[47]
***MZB1* (MZB1)**	Marginal zone B and B1 cell specific protein	51237	Regulation of apoptosis	Chronic phase Blast crisis	[44]
***NFKB1* (NFKB1)**	Nuclear factor kappa B subunit 1	4790	Regulator of NF-kB pathway	TKIs ^1^ resistance	[44]
***PDGFR*-β (PDGFRB)**	Platelet-derived growth factor receptor, beta polypeptide	100487523	Regulation of cell proliferation, survival, differentiation, chemotaxis, and migration	Chronic phase Blast crisis	[47]
***PTGS1* (PTGS1)**	Prostaglandin-endoperoxide synthase 1	5742	Drug metabolism; Regulation of cell proliferation	TKIs ^1^ resistance	[88]
***PTK2* (PTK2)**	Protein tyrosine kinase 2	5747	Regulation of cell growth and cell adhesion	Chronic phase Blast crisis	[47]
***PTKB2* (PTKB2)**	Protein tyrosine kinase 2 beta	2185	Calcium-induced regulation of ion channels	Chronic phase Blast crisis	[47]
***PTPN22* (PTPN22)**	Protein tyrosine phosphatase non-receptor type 22	26191	CBL function in the T-cell receptor signaling pathway	TKIs ^1^ resistance	[88]
***RGS2* (RGS2)**	Regulator of G protein signaling 2	5997	Regulator of myeloid differentiation	Chronic phase Blast crisis	[44]
***SKIL* (SKIL)**	SKI like proto-oncogene	6498	TGF-β pathway–regulation of cell growth and differentiation	TKIs ^1^ resistance	[44]
***SORBS3* (SORBS3)**	Sorbin and SH3 domain containing 3	10174	Cell adhesion	TKIs ^1^ resistance	[88]
***SQSTM1* (SQSTM1)**	Sequestosome 1	8878	Regulator of NF-kB pathway	TKIs ^1^ resistance	[44]
***SRC* (SRC)**	SRC proto-oncogene, non-receptor tyrosine kinase	6714	Regulation of cell growth	Chronic phase Blast crisis	[47]
***TGFB1* (TGFB1)**	Transforming growth factor beta 1	7040	Regulation of cell proliferation, differentiation, and growth	Chronic phase Blast crisis	[47]
***TGFB2* (TGFB2)**	Transforming growth factor beta 2	7042	Regulation of cell proliferation, differentiation, and growth	Chronic phase Blast crisis	[47]
***TNC* (TNC)**	Tenascin C	3371	Regulation of cell adhesion	TKIs ^1^ resistance	[88]
***TNFA* (TNF)**	Tumor necrosis factor	7124	Cytokine involved in inflammatory response. Cell proliferation and differentiation	Chronic phase Blast crisis	[47]
***TP53* (p53)**	Tumor protein p53	7157	Regulation of cell-cycle, apoptosis, and autophagy	Chronic phase Blast crisis Mutations correlated to poor prognosis	[46,47,48,49,50]
***VEGFA* (VEGFA)**	Vascular endothelial growth factor A	7422	Proliferation and migration of vascular endothelial cells	Chronic phase Blast crisis	[47]
***WTAP* (WTAP)**	Wilms tumor (*WT1*) associated protein	9589	Cell cycle regulation	TKIs ^1^ resistance	[44]

^1^ TKIs–tyrosine kinase inhibitors.

**Table 2:** miRNAs involved in CML phenotype.

miRNA	Expression in CML	Process Involved/Biological Relevance	References
**miRNA-21**	Upregulated	One of the most upregulated miRNAs in cancer, Implicated in drug resistance. Low levels at diagnosis are associated with an optimal response to TKI therapy. Biomarker of disease progression: expression increases from CP to BP. Inhibition leads to G1-phase arrest, growth inhibition, and apoptosis.	[149,150,151,152]
**miRNA-29b**	Downregulated	Expression level is negatively correlated with *MCL-1* and *BCL-2* expression. Downregulation involved in apoptosis evasion.	[153]
**miRNA-30a**	Downregulated	Inhibits autophagy, thus promoting IM cytotoxicity: upregulated in IM-responders. Expression levels are negatively correlated with Sokal score and BCR-ABL1 following treatment.	[154,155]
**miRNA-138**	Downregulated	Associated with downregulation of *BCR-ABL1*, inhibition of proliferation, and IM-induced apoptosis.	[156]
**miRNA-203**	Downregulated	Frequently silenced in CML (monoallelic loss and promotor hypermethylation). IM inducemiRNA-203 promoter demethylation: miRNA-203 upregulation decreases *BCR-ABL1* expression and reduces CML cells proliferation rate.	[157,158]
**miRNA-451**	Downregulated	Biomarker of disease progression: expression decreases from CP to BP. Reciprocal regulatory loop between miRNA-451 and *BCR-ABL1* Biomarker of prognosis and treatment response: involved in IM resistance, expression levels at diagnosis predict TKI response.	[149,159,160,161,162]
**miRNA-144/451**	Downregulated	Regulatory pathway between miRNA-144/451 and *c-MYC* Downregulated in IM resistant patients (c-*MYC* upregulated); Restoration of miRNA-144/451 expression levels sensitize CML cells to IM.	[163]
**miRNA-486-5p**	Downregulated	Valuable prognosis biomarker: higher miRNA-486-5p expression levels at diagnosis are associated with better prognosis and faster achievement of complete hematologic response to IM treatment.	[164]

TKI, Tyrosine Kinase Inhibitor; CP, chronic phase; BP, blast phase; *MCL-1*, myeloid cell leukemia sequence 1; *BCL*-2, B-cell lymphoma 2; CML, chronic myeloid leukemia; IM, imatinib.
